# Tunneled flaps reconstruction after facial tumor resection: Retrospective study and new forehead flap

**DOI:** 10.1016/j.jpra.2025.10.015

**Published:** 2025-10-17

**Authors:** Giuseppe Consorti, Giulio Cirignaco, Lisa Catarzi, Mariagrazia Paglianiti, Enrico Betti, Umberto Committeri, Paolo Balercia, Alberto Bianchi, Gabriele Monarchi

**Affiliations:** a Department of Clinical Specialistic and Dental Sciences, Marche Polytechnic University, Via Tronto 10/A, 60126, Ancona, Italy; b Division of Maxillofacial Surgery, Department of Neurological Sciences, Marche University Hospitals- Umberto I, Via Conca 71, Ancona, Italy; c Department of medicine, Section of maxillo-facial surgery, University of Siena, Viale Bracci, Siena, Italy; d Maxillofacial Surgery, Santa Maria Hospital, Unit, V.le Tristano di Joannuccio, 05100, Terni, Italy; e Maxillo Facial Surgery Unit, CHIRMED Department, University of Catania, AOU Policlinico San Marco, Catania, Italy; f Department of General Surgery and Medical-Surgical Specialties, University of Catania, Via Santa Sofia 78, 95123, Catania, Italy

**Keywords:** Tunneled flap, Facial reconstruction, Skin tumors, Facial aesthetic subunits, Maxillofacial surgery

## Abstract

**Background:**

Surgical defects of the face resulting from the removal of skin tumors can present significant challenges, particularly when reconstructive methods aim to maintain the integrity of the facial anatomical units. The face is divided into specific aesthetic units, each characterized by skin with uniform features such as thickness, subcutaneous tissue, and adhesion to underlying structures.

**Material and methods:**

From 2014 to 2024, 24 patients were treated with tunneled flaps for facial defects due skin tumors at the Maxillofacial Surgery department, University Hospital of Ancona, Italy.

**Results:**

In a study of 24 patients (15 males, 9 females; mean age 73), only 5 had a history of malignant facial skin tumors, with 3 presenting due to local recurrence. Lesions ranged in size from 19 × 15 mm to 30 × 24 mm. 10 patients had disease at the eyelid level and 14 at the nose level. The most common diagnosis was basal cell carcinoma (15 cases), followed by squamous cell carcinoma (7 cases) and Merkel cell carcinoma (2 cases). No lymph node or distant metastases were detected preoperatively. All patients had good or very good cosmetic outcomes, with no complications or recurrences observed during follow-up. No complication was observed.

**Conclusion:**

The tunneled pedicled flap is an excellent choice for reconstructing upper eyelid and nasal defects. This technique is easy to manage, can be completed in a single stage with satisfactory outcomes, and effectively hides donor site scars along natural facial lines. Moreover, it maintains facial symmetry and can be performed under local anesthesia, with or without sedation.

## Introduction

Surgical defects of the face, resulting from the removal of skin tumors of varying sizes and depths, can present significant challenges, particularly when using reconstructive methods that aim to preserve the anatomical units of the face.

Reconstructing facial defects necessitates a thorough understanding of both anatomical landmarks and facial units, as well as how to preserve the function and cosmetic appearance of the treated area. These facial units are defined by natural folds and boundaries, and they share similarities in color, texture, thickness, subcutaneous fat, mobility, and hair distribution.

To achieve minimally noticeable scars in facial reconstruction, it is essential to consider aesthetic units: the flap should be designed within the aesthetic unit containing the primary defect, and incisions should be placed along the boundaries of these units without crossing them.^1^

While numerous pedicled or island reconstructive flaps are described in the literature, the key consideration is their application for reconstructive purposes rather than mere knowledge of them. The characteristics of the surgical defect, such as its location and size, and the varying skin qualities among individuals are crucial factors.

The first subdivision of the aesthetic sectors of the face was proposed by Gonzalez-Ulloa^2^ with the introduction of 14 sectors; this subdivision was later expanded to 9 units and 16 subunits.^3^

The reconstruction of skin defects in the nasal and eyelid region has always represented an important surgical challenge as unfortunately they are areas of the face that are difficult to conceal and any non-optimal scarring results can greatly affect the patient's life.

To divide the face into aesthetic subunits, the classification made by Fattahi is very complete and exhaustive and includes the following units:1, Forehead unit (1A, central subunit; 1B, lateral subunit; 1C, eyebrow subunit); 2, nasal unit; 3, eye lid units (3A, lower lid unit; 3B, upper lid unit; 3C, lateral canthal subunit; 3D, medical canthal subunit); 4, cheek unit (4A, medial subunit; 4B, zygomatic subunit; 4C, lateral subunit; 4D, buccal subunit); 5, upper lip unit (5A, philtrum subunit; 5B, lateral subunit; 5C, mucosal subunit); 6, lower lip unit (6A, central subunit; 6B, mucosal subunit); 7, mental unit; 8, auricular unit; 9, neck unit.^3^^,^^4^

The anatomy of the nasal structure is categorized into nine subunits: the dorsum (7), consisting of the right and left dorsal sidewalls (8–9); the tip(1), comprising the right and left alar sidewalls (4–5), right and left alar bases(3–6), and the columella(2) according to Burget and Menick.^5^

As a singular feature in the face, its structure underscores the significant importance of preserving the subunits and their characteristics, including color, texture, and volume.^6^^,^^7^

In reconstructive endeavors, employing tunneled cutaneous and subcutaneous island flaps offers a viable alternative for addressing small facial surgical defects, particularly in regions requiring complex management, such as the upper and middle regions. This approach enables the attainment of excellent aesthetic and functional outcomes in a single surgical procedure, often under local anesthesia.

**Figure 1:**
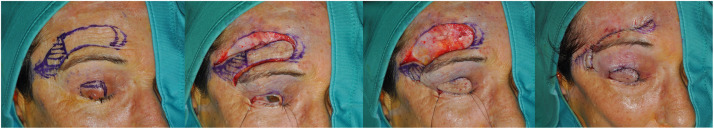
Figures show the evolution of a single patient from tumor excision to final outcome**.** a intraoperative drawing of the right paramedian forehead flap and drawing of the upper eyelid lesion; b skin incision and preparation of the pedicled flap; c transposition of the tunneled flap beneath the eyelid skin; d suture of recipient and donor site wounds.

This retrospective study aims to present local tunneled flaps as a viable alternative to the more well-known reconstructive flaps for facial skin defects with a specific focus on a new type of tunneled forehead flap for eyelid reconstruction (Figure 1a,b,c,d).

The following steps outline the technique in detail:

### Anesthesia and surgical field preparation

• The procedure can be performed under local anesthesia with or without sedation, depending on the defect size and patient conditions.
• A mixture of 2 % lidocaine with 1:100,000 epinephrine is used to achieve effective anesthesia and minimize intraoperative bleeding.
• The surgical area is disinfected with chlorhexidine or povidone-iodine, and sterile drapes are applied to isolate the operative field.

### Flap design and incision

• The flap is outlined using a dermographic pen, following the skin tension lines (Langer’s lines) to optimize aesthetic outcomes.
• The flap should be slightly larger than the defect to compensate for postoperative tissue contraction.
• Incisions are made using a #15 scalpel blade, taking care to avoid damage to underlying vascular and neural structures.

### Flap elevation and pedicle dissection

• The flap is elevated at either full-thickness or partial-thickness, depending on the reconstruction needs.
• The flap is raised from the periphery towards the center, preserving the vascularized pedicle.
• The pedicle is preserved at a minimum width of 10–20 mm to maintain adequate vascular supply.

### Creation of the subcutaneous tunnel

• A tunnel is created between the donor site and the recipient area using blunt dissection with curved scissors.
• The tunnel must be wide enough to allow the flap to pass without compression, reducing the risk of vascular compromise.

### Flap transposition and positioning

• The flap is carefully passed through the tunnel using atraumatic forceps.
• Care is taken to avoid excessive tension or rotation, keeping the pivot point below 90°
• The flap is positioned over the defect and adjusted for an optimal fit without excessive bulk or redundancy.
• The portion of the flap lying beneath the skin tunnel should be carefully deepithelialized to avoid complications.

### Flap fixation and donor site closure

• The flap is secured to the recipient site using 5–0 or 6–0 non-absorbable interrupted sutures, ensuring stability and minimizing tension.
• The donor site is closed by primary intention whenever possible. If closure is not feasible, it is left to heal by secondary intention with regular wound care.

### Postoperative management and follow-up

• The patient is monitored for 24–48 h to assess flap perfusion and detect early signs of ischemia or venous congestion.
• Dressings are changed every 2–3 days, and any signs of infection or delayed healing are closely monitored.
• Sutures are removed after 7–10 days, depending on the healing process.
• In cases where the flap exhibits excessive bulk or asymmetry, a refinement procedure with tissue debulking may be performed.
• Patients are followed up at 1, 3, 6, and 12 months to assess the final aesthetic and functional outcome and determine if further refinements are necessary.

This surgical technique, when executed with precision and attention to anatomical details, ensures excellent flap integration, minimal donor site morbidity, and optimal functional outcomes.

## Materials and methods

This retrospective study identified patients who underwent reconstruction with tunneled local flaps for skin tumors of the eyelid and nasal region, between January 2014 and December 2023 by a single Head and Neck (H&N) oncological surgeon at the Maxillofacial Surgery Department of Ancona.

We reviewed medical records and extracted data for all variables listed in Table 1. Medical records were analyzed to extract information on patient demographics, comorbidities, disease stage, treatment options, and surgery outcomes. For each patient, data were collected regarding the site of the disease, the initial staging, the size of the surgical defect and the complications and the long-term outcomes.

**Table 1:** Anamnestic history of patients.

Anamnestic History		
	YES	NO
Smoking habits	29 %	71 %
Cardiovascular diseases	53 %	47 %
• Hypertension	78 %	22 %
• Atrial fibrillation	33 %	67 %
• Ischemic cardiopathy	22 %	78 %
Anticoagulants and/or antiplatelet medication (before and after surgery)	53 %	47 %

The data were collected from the department’s internal database and from the Ormaweb surgery archiving program (Dedalus, Florence, IT).

Patients were evaluated at the preoperative time, at 1 postoperative week, after 2 postoperative weeks, at 1, 3, 6 and 12 postoperative months to analyze the healing process of the cutaneous wounds and any complications.

### Inclusion criteria

The specific criteria for performing tunneled flaps over other reconstructive options include:

1. Defect Location and Size
  ○ Nasal defects classified as types 2, 3, 4, and 5 according to Burget and Menick.^5^
  ○ Eyelid defects classified as types 3c and 3d based on Fattahi’s aesthetic subunit classification.^3^
  ○ Defects larger than 1.5 cm but smaller than 2.5 cm, where primary closure is not feasible and larger flaps or grafts are unnecessary.
2. Preservation of Aesthetic Subunits
  ○ When maintaining natural skin texture, thickness, and color is essential.
  ○ Avoidance of visible scarring across aesthetic unit boundaries.
  ○ Preference for local tissue use to blend with surrounding skin.
3. Minimally Invasive Approach
  ○ Suitable for patients requiring reconstruction under local anesthesia, with or without sedation.
  ○ Single-stage procedure with reduced surgical complexity compared to free flaps or staged reconstructions.
4. Superior Cosmetic and Functional Outcomes
  ○ Effective in maintaining facial symmetry and minimizing donor site morbidity.
  ○ Concealed donor site scars along natural facial lines.
  ○ Maintains vascular integrity with a well-preserved pedicle to reduce necrosis risk.
5. Patient Considerations
  ○ Patients who are compliant with post-operative care and follow-up.
  ○ Patients without prior free flap reconstruction or significant facial scarring that could interfere with flap viability.
  ○ Suitable for elderly patients or those with comorbidities where extensive surgery is contraindicated.

### Exclusion criteria

Patients who had undergone skin grafts, biosynthetic dermal matrix substitutes, or previous free flap reconstruction of the facial skin were not included in the study.

None of the patients in this study had facial scars that influenced the choice of reconstruction and all of them were followed up for a minimum of 12 months after the procedure.

In all patients, the surgical procedure consisted of tumor resection and reconstruction with tunneled flaps. The resection of the tumor tissue was performed with macroscopically healthy margins of 1 cm for basal-cell and squamous-cell carcinoma and 2 cm for Merkel cell carcinoma. The reconstruction consisted of local facial flaps transposed to reconstruct the tissue defect, following tunneling beneath the surrounding healthy tissue.

In both facial area for reconstruction of upper eyelid and non-surgical planning begins by demarcating the resection margins of the neoformation, following its removal the operation consists of steps, namely: flap design; tunnel creation beneath healthy tissue; flap transposition and surgical site reconstruction.

Tunneled island flaps, a modification of traditional island flaps, have been employed in facial reconstructive surgery. They are particularly useful for repairing deep defects in areas such as the inner canthus of the eye, the nasal dorsum, and the alae. In a tunneled flap procedure, a skin island of similar size to the defect is created near—but not directly adjacent to—it. The flap is fully incised around its surface but retains a subcutaneous pedicle, which remains long enough to allow the skin island to be mobilized through a subcutaneous tunnel from the donor site to the defect.

Among the flaps prepared and analyzed in this study, particular attention must be given to a new technique of tunneled pedicled forehead flap that is currently not present in the international scientific literature.

In the nasal reconstruction the width of the cutaneous flap that closes the external side of the nasal defect is at least 100 % of the alar defect because this slight difference recreates the natural convexity of the alar defect itself (Figure 4a,b).

### Follow up

After surgery, patients were asked to follow up 2 at 1 postoperative week, after 2 postoperative weeks to evaluate healing and to remove the sutures, at 1, 3, 6 and 12 postoperative months to analyze the complete healing process of the cutaneous wounds and any complications.

At each clinical visit, the status of the surgical wound was assessed until full healing was reported.

## Results

A total of 24 patients, comprising 15 males (62,5 %) and 9 females (37.5 %), with a mean age of 73 years (ranging from 46 to 98 years old), were treated in our department.

Only 5 patients had a remote pathological history positive for malignant facial skin tumors, and of these, 3 came to our attention due to local disease recurrence.

In terms of patients' medical history: 7 were smokers (29 %); 13 (53 %) had cardiovascular conditions such as hypertension, atrial fibrillation, or ischemic cardiopathy; and 13 (53 %) were prescribed anticoagulants/antiplatelet medication both before and after surgery (Table 1).

Out of the total number of patients, 10 patients had disease at the eyelid level and 14 at the nose level. 12 patients underwent treatment under local anesthesia alone, while 5 received local anesthesia combined with sedation. The size of lesion was from 19 × 15 mm to 30 × 24 mm with a mean size of 23,21 × 18,25.

**Figure 2:**
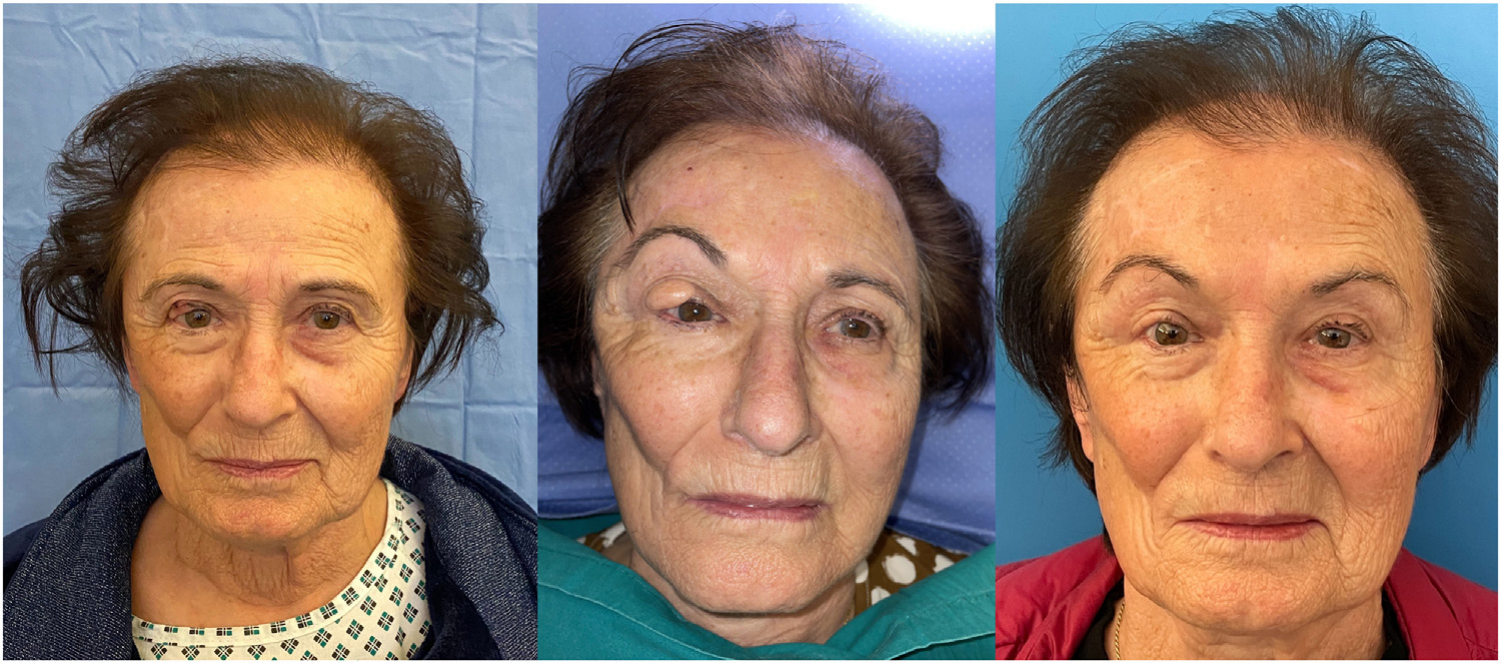
a Preoperative image of the second refinement surgery showing the lesion of the right upper eyelid; b Postoperative image displaying the reconstructive flap in excess relative to the remaining eyelid portion; c Image at the end of refinement procedures and reduction of the reconstructive flap.

**Figure 3:**
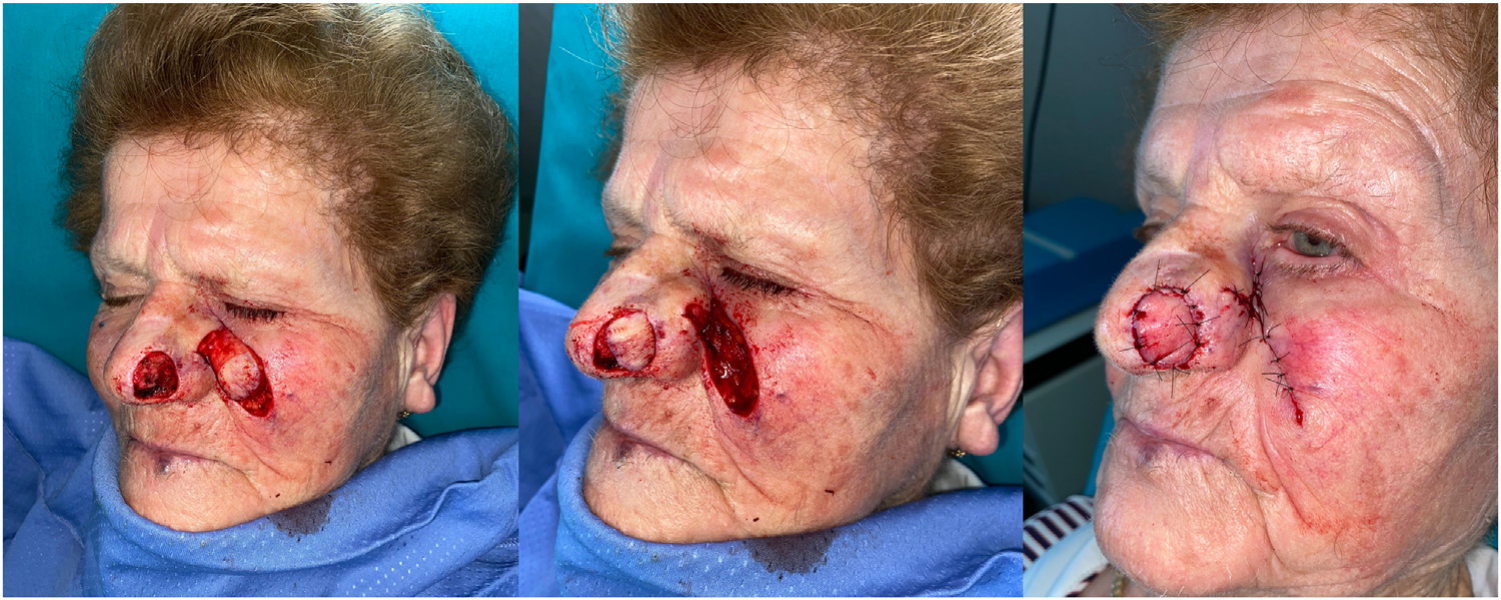
Figures show the evolution of a single patient from nasal skin tumor excision to final outcome. a Intraoperative image of the nasal defect and the left nasolabial flap; b transposition of the tunneled flap to reconstruct the tissue defect; C suture of recipient and donor site wounds.

**Figure 4:**
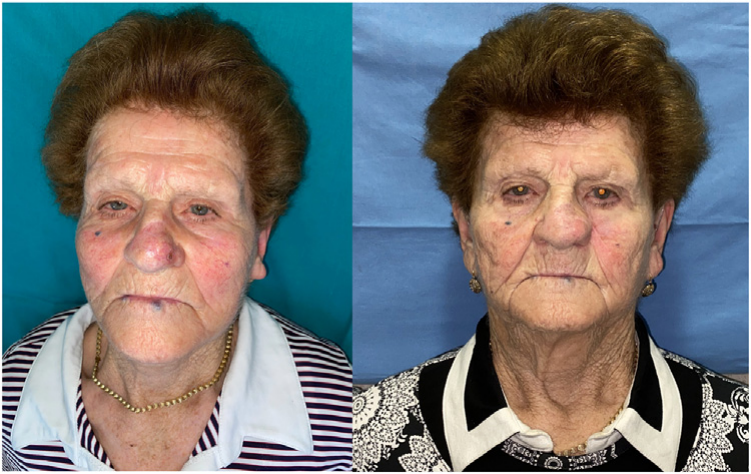
a Preoperative image showing the left paramedian nasal lesion; b Postoperative image at 3-month follow-up displaying excellent wound healing and nasal reconstruction.

The 9 patients suffering from malignant lesions of the upper eyelid (type 3c and 3d defects) were reconstructed by preparing the new tunneled paramedian forehead flap (Figure 1a,b,c,d) (Figure 2a,b,c). The 15 patients suffering from tissue defects of the nose (type 2, 3,4,5) secondary to the removal of malignant lesions were reconstructed using a tunneled nasolabial flap (Figure 3a-c and Figure 4a,b) (Table 2).

**Table 2:** Characteristics of the treated tumors.

Patients	Dimension (mm)	Histological type	Histological grading	Location of the surgical defect	Type of flap
1	19 × 15	BCC	G2	3d (Upper eyelid)	TFF
2	20 × 16	SCC	G1	3 (Nasal subunit)	TNF
3	28 × 15	BCC	G3	4(Nasal subunit)	TNF
4	21 × 17	SCC	G2	3c (Upper Eyelid)	TFF
5	26 × 20	SCC	G3	3(Nasal subunit)	TNF
6	30 × 21	BCC	G1	4(Nasal subunit)	TNF
7	25 × 18	BCC	G2	5(Nasal subunit)	TNF
8	23 × 20	BCC	G1	2(Nasal subunit)	TNF
9	19 × 15	MCC	G1	2(Nasal subunit)	TNF
10	25 × 17	BCC	G3	5(Nasal subunit)	TNF
11	20 × 15	BCC	G1	3c (Upper eyelid)	TFF
12	30 × 24	BCC	G3	3(Nasal subunit)	TNF
13	26 × 21	SCC	G1	4(Nasal subunit)	TNF
14	15 × 23	SCC	G2	3c (Upper eyelid)	TFF
15	23 × 18	SCC	G2	4(Nasal subunit)	TNF
16	25 × 17	BCC	G1	3c (Upper eyelid)	TFF
17	23 × 16	BCC	G1	3c (Upper Eyelid)	TFF
18	27 × 18	BCC	G1	2(Nasal subunit)	TNF
19	22 × 19	BCC	G2	3d(Upper eyelid)	TFF
20	23 × 20	BCC	G3	3(Nasal subunit)	TNF
21	21 × 19	SCC	G2	3c (Upper eyelid)	TFF
22	22 × 17	BCC	G1	3d (Upper eyelid)	TFF
23	24 × 16	MCC	G2	5(Nasal subunit)	TNF
24	20 × 21	BCC	G3	3c (Upper eyelid)	TFF

BCC, basal cell carcinoma; SCC, squamous cell carcinoma; MCC, Merkel cell carcinoma; TNF, tunneled nasolabial flap; TFF, tunneled frontal flap.

The definitive histological diagnosis of the resected lesions revealed basal cell carcinoma in 15 cases, squamous cell carcinoma in 7 cases, and Merkel cell carcinoma in 2 cases.

Concerning the grading of the malignant pathology removed, a G1 was found in the 42 % of cases, a G2 grading in the 33 % and a G3 in the 25 %.

Clinical and radiological involvement of cervical lymph nodes and distant metastases, at the preoperative stage, was not found in any patient (all cN0 and cM0 cases).

The cosmetic outcome, rated as good or very good by both patients and surgeons, was consistently achieved in all cases. Throughout both short and long-term follow-up periods, complications such as hematoma, infection, donor site morbidity, tumor recurrence, and facial nerve weakness/palsy were not observed. Additionally, all facial units were preserved, and surrounding tissues remained undistorted or displaced within their anatomical landmarks. All patients enrolled in the study were regularly monitored through follow-up appointments, and no instances of recurrence were observed throughout the follow-up period.

All patients demonstrated excellent healing at the surgical sites and achieved optimal aesthetic outcomes. There were no instances of partial or complete flap loss, as all island flaps healed successfully with satisfactory results.

## Discussion

Managing facial skin cancer can present challenges, particularly in the reconstruction of surgical defects. This reconstruction must adhere to fundamental principles: preserving the function of the reconstructed unit while prioritizing an optimal aesthetic outcome. A comprehensive understanding of anatomical landmarks and facial units is essential in this process.

Burget and Menick were the first to introduce a new analysis of facial units, particularly concerning the nasal surface. They described the nose as being composed of "slightly convex and concave surfaces separated by shallow valleys and ridges," which can be referred to as subunits.^5^

This new subdivision, introduced by Kirwan, offers a more comprehensive perspective on classification by incorporating planned surgeries within the head and neck region.^7^

Historically, reconstructing defects involving one or more aesthetic units has often relied on V-Y advancement island flaps,^8^ which have proven valuable, particularly in regions like the angle of the upper lip, cheek, and medial canthus.^9^ However, in some cases flaps such as the V-Y can have important advantages, particularly in terms of movement, as their design can sometimes allow for a single unit advance and rotations of up to 180° when used to fill particularly large defects.^9^

In these types of reconstructions, it is crucial to emphasize the importance of selecting flaps that best suit the specific subunit, as the flaps we have chosen—particularly the new technique—offer an excellent reconstructive solution both aesthetically and morphologically for the nose and eyelid.

Utilizing transposition pedicled flaps for facial defect reconstruction undoubtedly poses a discussion, particularly in addressing defects at the junctions between distinct anatomical units such as the nose, upper lip, upper cheek, and lower eyelid.^14^ This primary limitation is exacerbated by the shorter pedicle length, resulting in increased mobility and tension of the reconstructive flap.

Conversely, having a larger pedicle enclosed within a subcutaneous tunnel provides enhanced mobility. However, it must be of sufficient thickness to prevent potential necrosis or compromise of the flap, with a length-to-diameter ratio ideally at 3:1 or an absolute length of 1–1.5 cm^2^, while ensuring it is not overly wide to maintain mobility and prevent redundancy.^13^^,^^15^

In current literature on eyelid reconstruction, a limited number of local flaps are frequently described, with some of the most well-known being the Mustardè flap, the Tripier flap, ecc. This anatomical region demands highly precise reconstruction due to the crucial role of the eyelids in daily life. This necessity has driven the search for viable alternatives in eyelid reconstruction using local flaps.^16^^,^^17^

Various techniques are documented in the literature for upper eyelid reconstruction, with the general consensus being that the surgical approach should be tailored based on the quantity and quality of tissue requiring reconstruction.^18^^,^^19^

Upper eyelid defects are less common than inferior eyelid defects and necessitate reconstruction that preserves lid competence, thereby preventing corneal damage and ensuring an acceptable aesthetic outcome.^20^^,^^21^

Partial defects can be managed with advancement flaps like lateral myocutaneous advancement flaps.^10^^,^^22^ Full-thickness defects involving the tarsal can be addressed using various options, such as the galeal-pericranial flap released from the frontalis muscle and frontal bone,^11^ the inferior lid Cutler-Beard flap,^12^ or Mustarde's semimicircular lower lid flap.^11^ Nasal reconstruction offers different approaches, including the use of flaps such as the paramedian forehead tunneled flap or the merolabial flap.^6^

The new flap introduced by the author allows you to reconstruct >2/3 of the upper eyelid at full thickness. Offering minimal morbidity and reconstructed tissue quality.

The criteria for reconstructing a surgical defect on the nasal pyramid vary depending on the size. For defects under 1.5 cm, primary surgical closure may be feasible. Defects ranging from 1.5 cm to 2.5 cm are often addressed with local flaps. For defects exceeding 2.5 cm, various alternatives exist, including the paramedian forehead flap or an island tunneled flap.^6^^,^^23^

In the cases presented in our article, the tunneled flap has proven to be an effective option, enabling the reconstruction of the eyelid subunit with excellent aesthetic outcomes and complete functional restoration.

The authors created these types of flaps because they are less complex to prepare compared to other local, pedicled, or free flaps.

Immediate complications reported in the literature for tunneled flaps include hematoma, bleeding, infection, and partial flap necrosis,^13^^,^^24^ while long-term complications may involve lymphatic obstruction, scar hypertrophy, contracture, excess subcutaneous tissue, or the trapdoor effect commonly observed in transposition flaps, leading to a hypertrophic tissue deformity at the surgical site margins.^6^

Suggestions to mitigate this recognized complication include reducing the size of the islet by 20–25 %,^16^ subdominating the margins, creating a pedicle with the smallest possible base, and implementing post-operative massage.^17^

### Limitations of the study

Despite the promising results observed in this study, several limitations must be acknowledged. First, the study is retrospective in nature, which inherently limits the ability to establish causal relationships. Second, the sample size of 24 patients is relatively small, potentially restricting the generalizability of the findings to broader populations. Larger, multi-center studies would be beneficial to confirm these results. Additionally, all surgeries were performed by a single specialized head and neck oncological surgeon, which may introduce a bias related to surgical expertise. Another limitation is the lack of a control group undergoing alternative reconstructive techniques, which would allow for direct comparisons of efficacy, complications, and aesthetic outcomes. Future prospective studies with comparative analyses between different reconstructive approaches could provide more robust evidence regarding the advantages and limitations of tunneled flaps.

## Conclusion

We regard our reconstructive tunneled flap approach as highly versatile, enabling skin transfer from areas neighboring the surgical defect and thereby fostering outstanding aesthetic and functional outcomes. This technique offers numerous advantages: easy flap design and harvest, completion in a single surgical session with fewer intraoperative complications, scar concealment at cosmetic facial lines in the donor area, preservation of facial symmetry, and the option of performing the procedure under local anesthesia, with or without sedation.

The authors suggest these tunneled flaps for the 2,3,4, 5 subunits of the nose and for the 3c and 3d upper eyelid subunits for the excellent outcome, low morbidity, correct restoration of the affected subunits and easy preparation.

## Compliance with ethical standards

The authors, in the present study, adhered to the STROBE guidelines (http://www.strobe-statement.org/).

## Ethical approval

This study was approved by the Institutional Ethics Committee: Comitato Etico Regionale dell’ Umbria (CER) with approval number/protocol number 4755/24.

The research was conducted ethically, with all study procedures being performed in accordance with the requirements of the World Medical Association’s Declaration of Helsinki.

## Informed consent

Written informed consent was obtained from each participant/patient for study participation and data publication. Patients involved in the study provided cryptographic consent for the publication of photo.

No patient identifiable information was used. We declare that no ethical guidelines have been reached during the preparation and publication of this study

## Funding

The authors report no involvement in the research by the sponsor that could have influenced the outcome of this work.

## Declaration of generative AI in scientific writing

The authors declare that they have not used artificial intelligence programs to write this article.

## Authors’ contributions

All authors contributed equally to the manuscript and read and approved the final version of the manuscript.

## Declaration of competing interest

The authors certify that there is no conflict of interest with any financial organization regarding the material discussed in the manuscript.
